# Pneumothorax In Covid-19 Pneumonia: A case series

**DOI:** 10.1016/j.rmcr.2020.101265

**Published:** 2020-10-21

**Authors:** Mansoor Hameed, Wasim Jamal, Muhammad Yousaf, Merlin Thomas, Irfan Ul Haq, Shakeel Ahmed, Mushtaq Ahmad, Mohamad Khatib

**Affiliations:** a Hamad General Hospital, Hamad Medical Corporation, Doha, Qatar; b Hazm Mebaireek Hospital, Hamad Medical Corporation, Doha, Qatar; c Weill Cornell Medicine-Qatar, Cornell University, Qatar

**Keywords:** Pneumothorax. covid, 19. computed tomography. SARS-CoV −2

## Abstract

**Background:**

Coronavirus disease 2019 (Covid-19) is an infectious disease caused by severe acute respiratory syndrome coronavirus 2 (SARS-CoV-2). It mainly affects the lungs and common symptoms are fever, cough and shortness of breath. Pneumothorax has been noted to complicate Covid-19 cases requiring hospital admission, however the exact incidence and risk factors are still unknown

**Discussion:**

We present a series of 3 cases of primary spontaneous pneumothorax with Covid-19 pneumonia. All cases in our series did not require positive pressure ventilation and none had any pre-existing lung disease. All were never smokers and had favourable outcomes despite having severe Covid-19 with a pneumothorax during the course of the disease. In our literature review we discuss several plausible mechanisms and risk factors resulting in a pneumothorax with Covid-19.

**Conclusion:**

Our cases are a reminder that an acute deterioration with hypoxia in a Covid-19 patient could indicate a pneumothorax. Pneumothorax is one of the reported complications in Covid-19 and clinician vigilance is required during assessment of patients, as both share the common symptom of breathlessness and therefore can mimic each other.

## 1 Introduction

Coronavirus disease 2019 (COVID-19) is a communicable disease caused by severe acute respiratory syndrome coronavirus 2 (SARS-CoV-2). It started in December 2019 in Wuhan, China and the virus was first identified on Jan 6, 2020 [1]. Since then COVID-19 has spread globally, resulting in an on-going pandemic. SARS-CoV-2 is a single-stranded RNA virus, and bats are considered the most likely zoonotic source [2]. The most common symptoms of COVID-19 are fever, cough and shortness of breath. Real time–reverse transcription polymerase chain reaction (rRT-PCR) from a nasopharyngeal swab is diagnostic.

Radiological imaging plays an important role in the diagnosis and follow up of Covid-19 pneumonia [3]. Common computerized tomography (CT) findings in COVID-19 are of patchy ground-glass opacities with a peripheral or posterior distribution, mainly involving the lower lobes [4]. Pleural effusion, pericardial effusion, lymphadenopathy, cavitation, CT halo sign, and pneumothorax are some of the uncommon but possible findings seen with disease progression [5].

Pneumothorax has been noted to complicate cases of COVID-19 requiring hospital admission. We present here three cases of pneumothorax associated with Covid-19 in non-intubated patients. One of these patient's had two distinct episodes of pneumothorax, occurring bilaterally in sequential fashion. All three patients were demographically atypical being lifelong non-smokers, of average height and with no known pre-existing lung disease.

## 2 Case presentations

### 2.1 Case 1

A 49 years old gentleman confirmed to have Covid-19 by RT-PCR five days ago, was brought in via ambulance to the emergency department from quarantine with worsening cough, shortness of breath and a high-grade fever. He was a never smoker and a driver by profession. His past medical history was unremarkable. On examination, he was tachypnoeic with a respiratory rate of 22 breaths/min, febrile at 39.1 °C, normotensive at 115/65 mmHg and hypoxic with oxygen saturations of 85% on room air. General physical examination was unremarkable, his height was 161 cm with a weight of 66 kg and body mass index (BMI) of 25.5. Chest examination revealed bilateral basal crepitations with the chest radiograph showing bilateral lower zone infiltrates consistent with a diagnosis of Covid-19 pneumonia. Notable lab investigations included a white blood cell count (WBC) of 10.2 × 10^3^/μL with a lymphocyte count of 0.8 × 10^3^/μL, D-dimer of 1.47 mg/L (peak 2.96), CRP of 133 mg/L, peak Lactate Dehydrogenase (LDH) of 436 U/L and a ferritin of 8352 mcg/L. He required 10 L of oxygen to maintain oxygen saturation of 95% and was treated initially as per the local guidelines for Covid-19 and later received IV tocilizumab, convalescent plasma and IV methylprednisolone in view of risk of rapid deterioration. He gradually improved and was weaned off of oxygen over a period of 12 days without needing mechanical ventilation. A day later he complained of right sided chest pain and increasing shortness of breath requiring 5 L of oxygen to maintain saturations of 95%. Chest x-ray identified a large right sided pneumothorax with mediastinal shift towards the left. (Fig.1a) A right sided intercoastal chest drain was inserted. After another 5 days he complained of left sided chest pain and a chest radiograph this time confirmed a large left sided pneumothorax (Fig. 1b). Subsequently a left sided intercostal chest tube was inserted. A CT chest was performed which showed bilateral pneumatoceles/bullous disease with a pneumatocele/bullae in right and left lower lobes respectively (Fig. 1c). Further investigation revealed a normal serum Alpha −1 antitrypsin level. The right lung gradually expanded and the drain was removed after a period of two weeks while the left sided chest tube remained in situ for 16 days due to a persistent air leak until lung expansion whilst awaiting surgical intervention.

**Fig. 1a:**
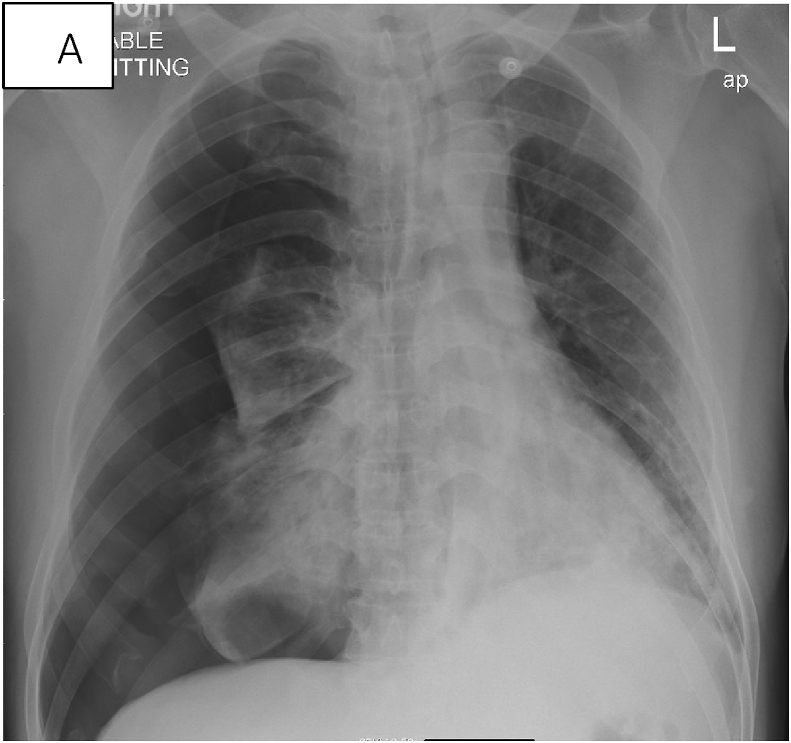
Large right-sided pneumothorax with mediastinal shift towards left.

**Fig. 1b:**
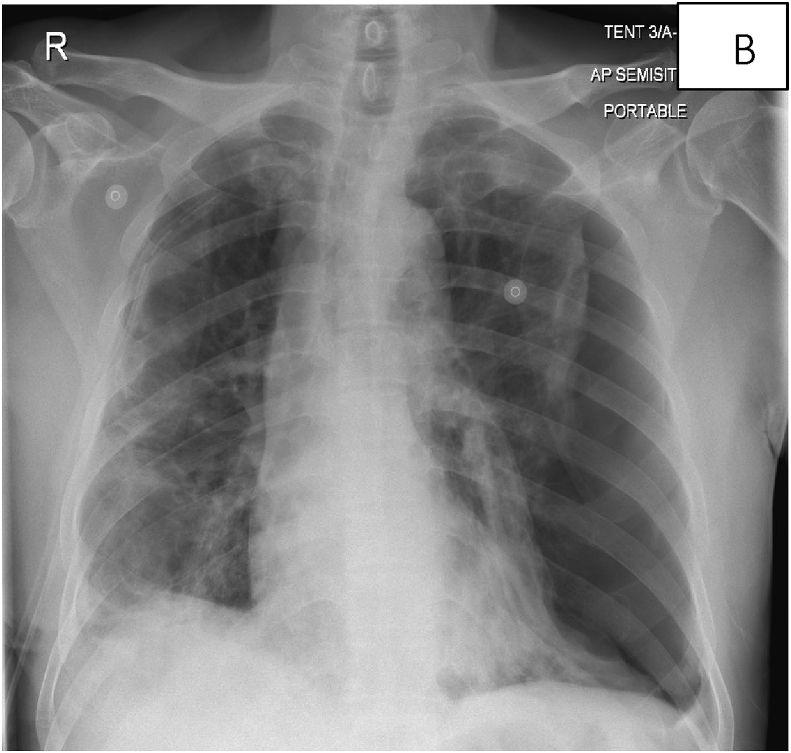
Left-sided pneumothorax. Right-sided chest tube in situ.

**Fig. 1c:**
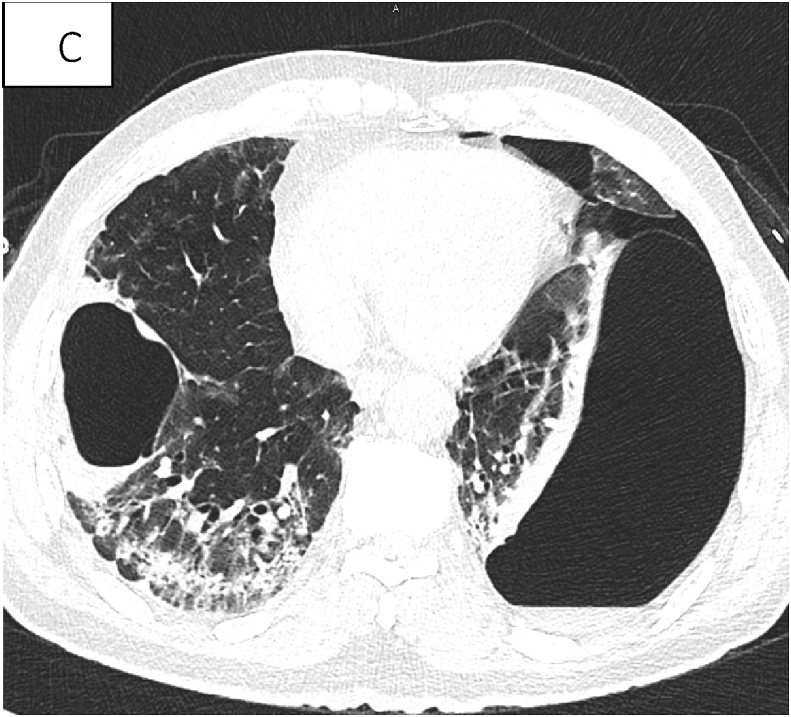
Right-sided pneumatocele/bullae seen with associated right lower lobe ground glass opacities; and left sided pneumothorax and pneumatocele.

### 2.2 Case 2

A 34 years old Nepalese man with no previous illnesses, presented to the emergency department with a 5 days history of fever, productive cough, shortness of breath, diarrhoea, generalized fatigue and myalgia. On admission he was febrile at 39.3 °C with a respiratory rate of 35 breaths/min. He required 10 L of oxygen to maintain saturations of 94%. He was a never smoker and his chest examination revealed bilateral crepitations. His height was 175cm with a weight of 92kg (BMI 30). Blood workup showed a CRP of 44 mg/L, D dimer 0.67mg/L (peak 1.41 mg/L), WBC 7.1 × 10^3^/μL, lymphocyte count of 1 × 10^3^/μL. He tested positive for Covid-19 by rRT-PCR with a cyclic threshold of 25.7. His CXR revealed bilateral lower zone non-homogenous infiltrates (Fig. 2A). He was commenced on treatment in accordance with the local hospital guidelines. He improved gradually and was weaned off oxygen over the next few days, however, on Day 7 he developed a right sided pleuritic pain and worsening shortness of breath with stable vital signs and oxygen saturations. A CT chest was requested which revealed a large right sided pneumothorax, consolidation of the right basal segments, and multifocal bilateral ground glass opacities in both lungs. (Fig. 2B). Subsequently a chest drain on the right side was inserted, resulting in full resolution of the pneumothorax in 6 days when the drain was removed and the patient was discharged home with a follow up. Of note was the fact that the patient gave a history of similar symptoms with ‘air in his chest’ ipsilaterally 3 years ago, managed with simple aspiration in his home country.

**Fig. 2a:**
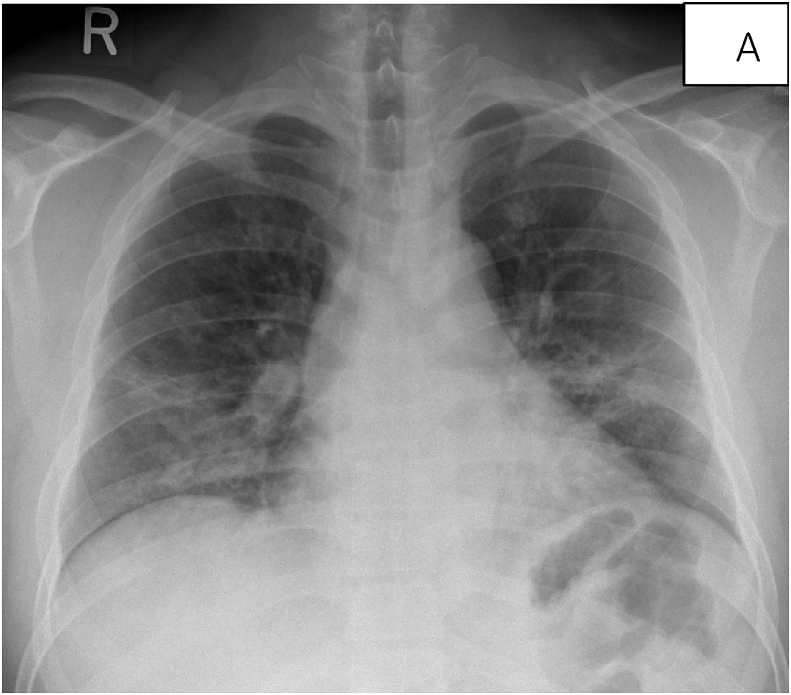
Chest radiograph: Bilateral lower and mid zone infiltrates.

**Fig. 2b:**
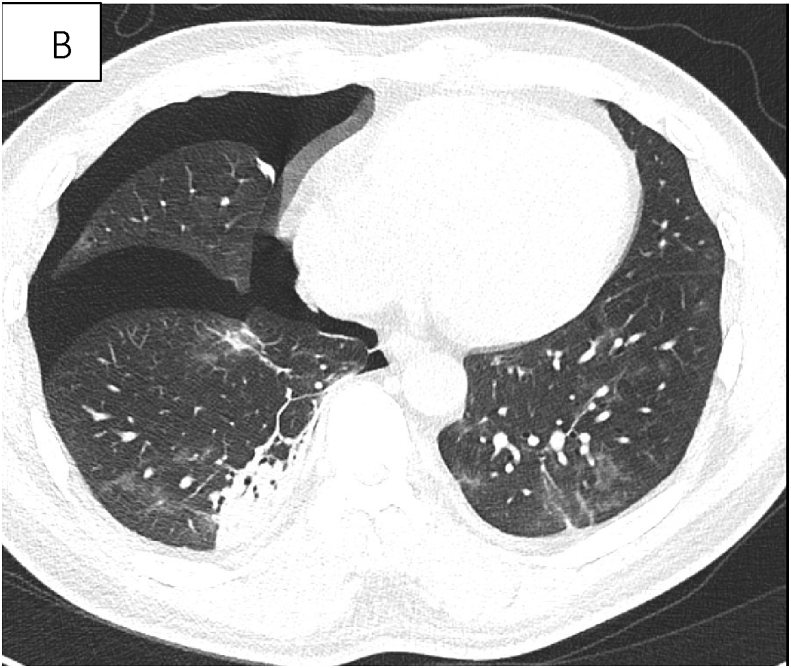
CT chest: Right pneumothorax with multifocal ground glass opacities and segmental consolidation of right lower lobe.

### 2.3 Case 3

A 47 years old Filipino man, presented to the hospital with a week's history of worsening shortness of breath, dry cough, fever and malaise. He was a never smoker and his past medical history was unremarkable. On examination he was found to be febrile at 39.6 °C with oxygen saturations of 97% on room air, respiratory rate of 16 breaths/min and BP 110/72 mmHg. His height was 168cm with a weight of 62kg (BMI 22). Chest examination revealed bilateral crackles and the CXR demonstrated bilateral patchy consolidation. His blood tests revealed a CRP of 76 mg/L (peak 311) ferritin 9619 mcg/L, D-dimer of 2.35 mg/L (peak 2.68) and WBC of 3.9 × 10^3^/μL with a lymphocyte count of 0.47 × 10^3^/μL and LDH of 812 U/L. A clinical diagnosis of COVID-19 pneumonia was made and he was commenced on treatment in accordance with the local guidelines. The following day his rRT-PCR for Covid-19 returned positive. Over the next few days there was a gradual clinical deterioration with worsening oxygen requirements of 15 L and he was also started on steroids. Although he improved gradually over the next 4 weeks without needing mechanical ventilation, he remained oxygen dependent needing 2litres persistently. In order to investigate his persistent hypoxia a CTPA was done showing no pulmonary embolism, but bilateral ground glass changes consistent with Covid-19 Infection. An incidental finding of a small right-sided pneumothorax was also noted (Fig. 3A). Due to the small size of the pneumothorax, it was managed conservatively. Subsequent chest x rays showed resolution and a HRCT chest on day 55 (Fig. 3B) showed no parenchymal abnormalities. He was discharged on 1L of home oxygen with a plan to review in the respiratory clinic in 6 weeks' time.

**Fig. 3a:**
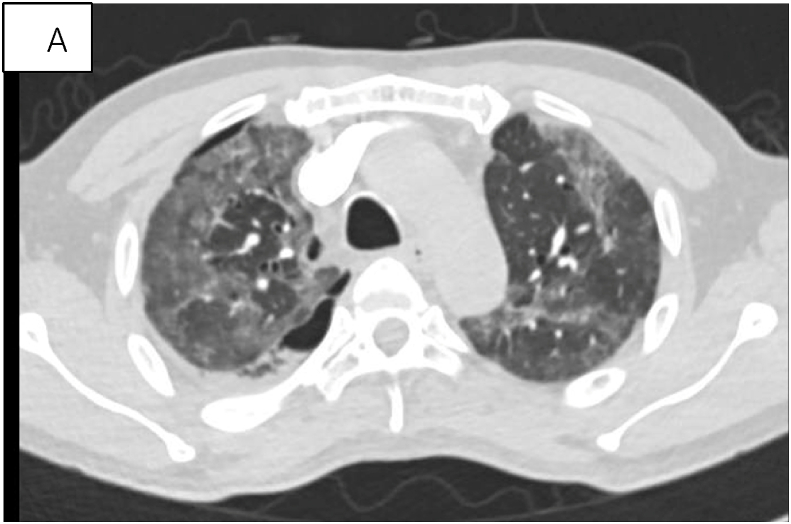
CTPA. Small right-sided pneumothorax. Bilateral ground glass opacities.

**Fig. 3b:**
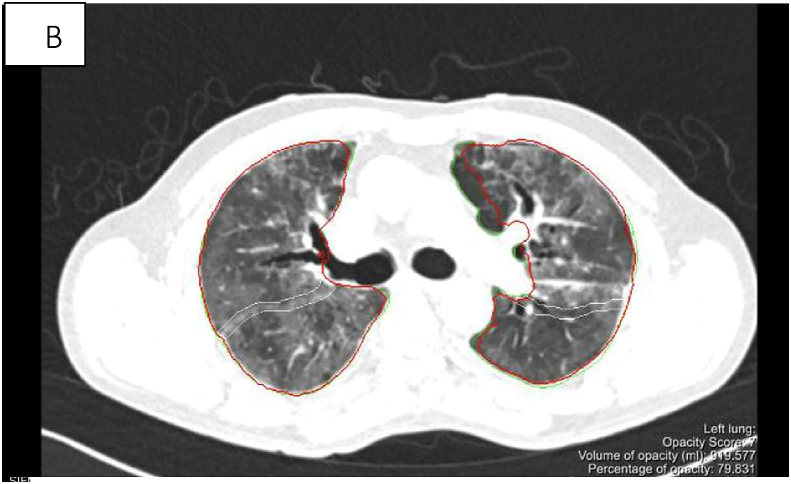
HRCT Chest. Resolution of previously noted pneumothorax. Bilateral ground glass changes.

## 3 Discussion

A pneumothorax is defined as air in the pleural space and can be classified as spontaneous (primary or secondary) or traumatic. Traumatic pneumothorax results from trauma including iatrogenic cases caused during procedures such as pacemaker insertions, while secondary spontaneous pneumothorax (SSP) occurs due to an underlying lung disease. Primary spontaneous pneumothorax (PSP) however, by definition, occurs in patients with no associated lung disease. Indeed, a finding of abnormal pleura however is very common even in PSP patients if looked for carefully, and include blebs and bullae, which are otherwise known as emphysema-like changes [6]. Although in all our patients we did not have any previous imaging to confirm underlying lung disease or bullae, we also did not have any reasons to suspect this based on the history and clinical examination except in one case where there was history of a previous ipsilateral pneumothorax. However even in that case cross sectional imaging did not reveal any radiological changes commonly associated with SSP.

While causality cannot be established through a case series between Covid-19 and pneumothorax, however our series adds to the previously published case series of pneumothorax in Covid-19 [15] adding to the weight of this being an associated complication of Covid-19 rather than being merely coincidental. Explaining the relationship between pneumothorax and Covid-19 is challenging with multiple possible mechanisms underlying this relationship. Pneumatoceles or cysts in patients with COVID-19 are described in the literature, even in patients not requiring positive pressure ventilation. This means that barotrauma associated with positive pressure ventilation alone cannot account for the cyst formation, which may contribute to the likelihood of developing a secondary pneumothorax [[7], [8], [9]]. Our cases series agrees with this hypothesis as none of our cases were mechanically ventilated ruling out barotrauma as the possible cause. Case 1 in our series however did show evidence of pneumatocele/cysts on cross-sectional imaging bilaterally, raising the possibility of their rupture leading to the pneumothorax as the possible mechanism, especially as he had two sequential pneumothorax bilaterally. Other possible causative factors for a pneumothorax in COVID-19 may be persistent coughing resulting in an increased intrathoracic pressure in the presence of underlying pleural abnormalities or alveolar damage from COVID-19 pneumonia related inflammation or ischaemic parenchymal damage [10].

In a case series of 6 patients with SARS and pneumothorax from Hong Kong, administration of methylprednisolone was thought to affect lung healing and the presence of a higher peak serum LDH and peripheral leucocyte count was postulated to depict a greater extent of lung injury thus raising the risk of a pneumothorax [11]. In our series all cases received steroids and had a high peak leucocyte count and LDH. Ferritin which is an acute phase reactant was also higher in 2 cases while one patient did not have it checked [Table 2].

Our case series call to attention the importance of considering pneumothorax as a differential in an acutely deteriorating patient with persistent hypoxia in Covid-19. The other important differential to consider is the possibility of a pulmonary embolism as several studies and case reports highlight its association with COVID-19 [12]. Pulmonary embolism related infarction can also result in parenchymal cavitation with subsequent pleural rupture leading to a pneumothorax however none of our patients had any clinical or radiological reasons to suspect this, although only the last case had a dedicated CTPA with the others having had non contrast CTs. The significance of identifying such secondary pathologies in COVID-19 is vital as the treatment required is very different, with possible life-threatening consequences in case the incorrect diagnosis or management is initiated. The fact that clinicians may also not regularly auscultate the chest given the concerns of exposure to SARS CoV-2, can further complicate a timely diagnosis and initiation of an appropriate management plan. Point of care ultrasound here can play a role in achieving a quicker diagnosis of pneumothorax if available by the bedside.

Development of pneumothorax in coronavirus infection has been thought of as a poor prognostic marker [11,14]. However, none of our cases had an adverse outcome [Table 1]. Our case series hence aligns with the largest multicentre case series published till date on pneumothorax in COVID-19 [15], highlighting the need to avoid a nihilistic approach in such cases and to pursue active management when clinically possible.

**Table 1:** Demographics, clinical characteristics and outcomes of the cases with pneumothorax and COVID-19 infection.

	Age/Gender	Height (cm)	Weight (Kg)	Symptoms on presentation	Risk factors for pneumothorax	Required Intensive care unit admission	Chest Tube	Duration of Hospital stay (Days)	Outcome
Case 1	49/M	161	66	Shortness of breath	None	No	Yes	35	Survived
Case 2	34/M	175	92	Fever, productive cough, shortness of breath, diarrhoea and generalized tiredness	Previous Ipsilateral Pneumothorax	No	Yes	21	Survived
Case 3	47/M	168	62	Shortness of breath, dry cough, fever and malaise	None	No	No	60	Survived

**Table 2:** Laboratory and Imaging characteristics of the cases.

	WBC (10^3^/μL)^a^	Lymphocyte (x 10^3^/μL)^a^	CRP (mg/L)^a^	Ferritin	LDH (U/L)^a^	D-Dimer (mg/L)^a^	First CXR	CT chest	Size Of Pneumothorax
Case 1	10.2 (peak 15.6)	0.8	133	8352	436	1.47 (peak 2.96)	Bilateral lower and mid zones infiltrates	Right sided pneumatocele/bullae with associated right lower lobe ground glass opacities; and left sided pneumothorax and pneumatocele.	Large
Case 2	7.1 (peak 14.3)	1.0	44	Not done	Not done	0.67 (peak 1.41)	Bilateral lower zones infiltrates	Large right sided pneumothorax, consolidation of the right basal segments, and multifocal bilateral ground glass opacities in both lungs.	Large
Case 3	3.9 (peak 11.0)	0.47	76 (peak 311)	9619	812	2.35 (peak 2.68)	Bilateral patchy infiltrates and consolidation	Small right-sided pneumothorax. Bilateral ground glass changes.	Small

a Reference values: WBC = 4–10 × 10^3^/μL; Lymphocytes = 1–3 x 103/μL; LDH = 135–225 U/L; ferritin = 30–553 μg/L; D-Dimer = 0.00–0.49 mg/L.

Management of pneumothorax in COVID-19 patients necessitating a chest drain based on the British thoracic society's (BTS) consensus guidelines, requires that this be undertaken in Level 1 PPE (surgical mask, visor, gown and gloves). BTS further recommends that bubbling chest drains should be considered for strategies to minimise droplet exposure via the chest drain circuit. This can be achieved by connecting the chest drain to wall suction (even in cases where suction is not normally indicated but set at a very low level such as 5cmH2O) thereby creating a closed system or by installing a viral filter onto the suction port of a Rocket chest drain bottle. Digital drain circuits are an alternative method of reducing risk of droplet spread, but they do not contain a viral filter [13].

## 4 Conclusion

Our case series highlights and consolidates previous reports of pneumothorax as a complication of COVID-19 pneumonia. Pneumothorax may develop in COVID-19 pneumonia due to multiple plausible mechanisms. These may include parenchymal injury, inflammation, ischemia, infarction, cough and pneumatocele rupture but a causality cannot be established based on a case series. Both COVID-19 pneumonia and pneumothorax share a non-specific but common symptom of dyspnoea. Our cases are a reminder that an acute deterioration with a rapid oxygen desaturation in a Covid-19 patient could indicate a pneumothorax. Clinicians should therefore be aware that a pneumothorax can be observed within the radiological and physiological manifestations of Covid-19 pneumonia and it may lead to an increase in mortality or morbidity.

## Financial Support

The publication of this article was funded by the Qatar National Library.

## Declaration of competing interest

The authors declare that they have no known competing financial interests or personal relationships that could have appeared to influence the work reported in this paper.
